# *CLDN1* regulates trophoblast apoptosis and proliferation in preeclampsia

**DOI:** 10.1530/REP-20-0677

**Published:** 2021-03-30

**Authors:** Yu-chen Zhang, Xiao-li Qin, Xiao-ling Ma, Hui-qin Mo, Shi Qin, Cheng-xi Zhang, Xiao-wei Wei, Xue-qing Liu, Yan Zhang, Fu-ju Tian, Yi Lin

**Affiliations:** 1 Shanghai Key Laboratory of Embryo Original Diseases, The International Peace Maternity & Child Health Hospital, Shanghai Jiao Tong University School of Medicine, Shanghai, China; 2 The International Peace Maternity & Child Health Hospital, Shanghai Jiao Tong University School of Medicine, Shanghai, China; 3 Department of Obstetrics and Gynecology, Shanghai First Maternity and Infant Hospital, Tongji University of Medicine, Shanghai, China; 4 Department of Obstetrics and Gynecology, Renmin Hospital of Wuhan University, Wuhan, Hu Bei, China

## Abstract

Preeclampsia is a gestational hypertensive disease; however, preeclampsia remains poorly understood. Bioinformatics analysis was applied to find novel genes involved in the pathogenesis of preeclampsia and identified *CLDN1* as one of the most differentially expressed genes when comparing patients with preeclampsia and healthy controls. The results of the qRT-PCR, Western blotting and immunohistochemistry experiments demonstrated that *CLDN1* was significantly downregulated in the chorionic villi in samples from patients with preeclampsia. Furthermore, knockdown of *CLDN1* in HTR-8/SVneo cells resulted in the inhibition of proliferation and induction of apoptosis, and overexpression of *CLDN1* reversed these effects. In addition, RNA-seq assays demonstrated that the gene *BIRC3* is potentially downstream of *CLDN1* and is involved in the regulation of apoptosis. Knockdown of *CLDN1* confirmed that the expression level of *BIRC3* was obviously decreased and was associated with a significant increase in cleaved PARP. Interestingly, the apoptotic effect in *CLDN1* knockdown cells was rescued after *BIRC3* overexpression. Overall, these results indicate that a decrease in *CLDN1* inhibits *BIRC3* expression and increases cleaved PARP levels thus participating in the pathogenesis of preeclampsia.

## Introduction

Preeclampsia (PE) is a complication of pregnancy characterised by high blood pressure, and it leads to a series of unpleasant outcomes, such as placental abruption, intrauterine growth retardation and preterm birth (Mol *et al.* 2016). According to the 2019 ACOG Practice Bulletin, PE is diagnosed based on abnormal blood pressure after 20 weeks of gestation with nonobligatory confirmation by the presence of proteinuria (ACOG 2019). PE can be accompanied by multiorgan dysfunction, including thrombocytopenia, renal insufficiency, impaired liver function and nervous system dysfunction. To date, many studies have investigated the mechanism of this gestational hypertensive disorder; however, detailed molecular mechanism of the disease is unknown. A widely accepted theory describes the pathogenesis of the disease as a ‘two stage’ process: abnormal placentation occurring in the first-trimester of pregnancy and consequent maternal syndrome in the second- or third-trimester of pregnancy. Under physiological circumstances, endovascular extravillous trophoblast entered inside spiral arteries vessel wall after interstitial extravillous trophoblast invasion; shallow invasion of endovascular extravillous trophoblast results in insufficient conversion of spiral arteries and the consequent poor placentation (Turco & Moffett 2019, Staff *et al.* 2020). Insufficient trophoblast invasion results in incomplete spiral artery remodelling and consequent reduced placental perfusion, and decidual vasculopathy and poor uterine decidualisation are potential reasons leading to placental insufficiency (Fisher 2015, Phipps *et al.* 2019, Rana *et al.* 2019). Increasing evidence has demonstrated that both foetal and maternal factors contribute to abnormal placentation. The development of technologies and numerous tactics assisting with the exploration of the mechanism of the disease can be used to identify additional contributing factors.

Claudin-1 is encoded by the *CLDN1* gene and is the first member of the claudin family. CLDN1 is a component of the tight junction complex, which controls epithelial homeostasis by regulating paracellular movement of the molecules (Garcia-Hernandez *et al.* 2017). Dysfunction of *CLDN1* is involved in the pathogenesis of multiple diseases. For example, *CLDN1*-deficient mice die soon after birth due to cutaneous dehydration (Furuse *et al.* 2002). Patients with atopic dermatitis show reduced expression of *CLDN1* in the nonlesional epidermis (De Benedetto *et al.* 2011). A recent study demonstrated that *CLDN1* plays a key role in cutaneous wound healing (Volksdorf *et al.* 2017). In addition, *CLDN1* is widely expressed in the intestinal epithelium; increased expression of *CLDN1* is observed in intestinal inflammatory disorders, such as Crohn’s disease and ulcerative colitis (Garcia-Hernandez *et al.* 2017). Abnormal expression levels of *CLDN1* were also detected in malignant tumours (Stache *et al.* 2014, Huang *et al.* 2015, Zhang *et al.* 2016). Diminished expression of *CLDN1* has been detected in PE placentas in other studies (Lievano *et al.* 2006, Pirinen & Soini 2014); however, the mechanism behind this phenotype has not been demonstrated.

In the current study, we confirmed that the expression of *CLDN1* was downregulated in the placental villus tissue in PE compared to that in uncomplicated pregnancy and identified a novel function of *CLDN1* in trophoblasts. Moreover, inhibition of *CLDN1* expression results in an increase in cleaved PARP via downregulation of *BIRC3* expression.

## Materials and methods

### GEO data analysis

GEO datasets (GSE66273, GSE75010, GSE96984 and GSE114691) were downloaded from the GEO database (https://www.ncbi.nlm.nih.gov/geo/). PCA was initially used to evaluate the expression profiles between the two groups (Supplementary Fig. 1, see section on supplementary materials given at the end of this article), and the expression matrix was validated prior to subsequent analysis. Differential genes based on the array data were analysed using the limma package for R. For RNA-seq data, transcripts were filtered and preprocessed using the voom function before differential gene identification using the limma package. Genes with adjusted *P* values <0.05 (BH method) and absolute log_2_ FC >1 were identified as differentially expressed genes.

**Figure 1:**
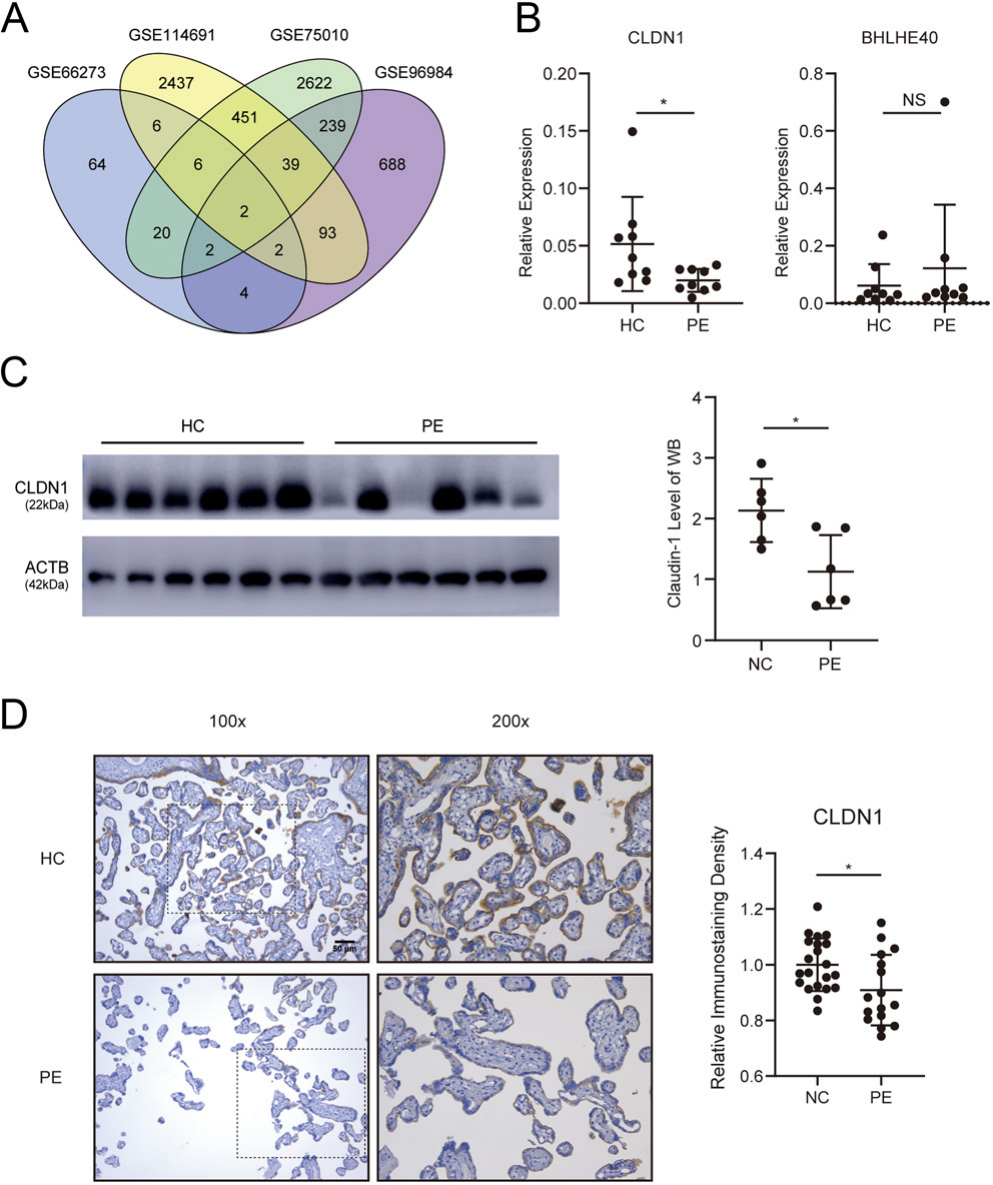
*CLDN1* is decreased in the placental villus tissue of patients with preeclampsia. (A) Bioinformatics analysis of differentially expressed genes from the GEO datasets. (B) *CLDN1* expression in placental villi from preeclampsia (PE) patients or healthy controls (HCs) was determined using qRT-PCR (*n* = 9). (C) Western blotting (*n* = 6) of the expression of CLDN1 in preeclampsia patients. (D) Images of IHC staining for CLDN1 (*n* = 21 in the HC group, *n*  = 16 in the PE group) and relative immunostaining density scores in the PE and HC groups quantified using Image-Pro Plus 6.0. A Mann–Whitney test was used in (B), and a Student’s *t*-test was used in (C) and (D), and the data are presented as the mean ± s.d. *P < 0.05, NS, not significant.

### Patient information

Last trimester placental villus tissue was collected at the International Peace Maternity & Child Health Hospital, China Welfare Institute, Shanghai Jiao Tong University School of Medicine. Patients with the following diagnoses were excluded: (1) foetal chromosomal malformation; (2) pregnancy complicated with endocrine or metabolic diseases (e.g. gestational diabetes mellitus, hypothyroidism and hyperthyroidism); (3) chorioamnionitis; (4) deleterious physical or chemical exposure during gestation; and (5) other diseases associated with pregnancy complications (e.g. previously diagnosed hypertension and renal diseases). A total of 25 preeclampsia (PE) patients and 27 subjects with uncomplicated pregnancies at 32–41 gestational weeks were included based on the diagnostic criteria for PE of the 2019 ACOG Practice Bulletin (Table 1 and Supplementary Table 2). All patients were fully informed, and consent was obtained in a written form. Ethics approval for this study was granted by the Medical Ethics Committee of the International Peace Maternity & Child Health Hospital of the China Welfare Institute, Shanghai, China.

**Table 1:** Clinical information of patients included in this study. Data were presented as the mean ± s.d. and statistical analysis was done performed using GraphPad Prism 8. A Student’s *t*-test or Mann–Whitney test was used to compare two groups.

Characteristics	Healthy controls (*n* = 27)	Preeclampsia (*n* = 25)
Maternal age, years	30.19 ± 3.076	33.52 ± 4.656**
Gestational day	273.3 ± 12.14	256.1 ± 16.70****
Systolic blood pressure, mmHg	117.1 ± 9.698	150.5 ± 15.12****
Diastolic blood pressure, mmHg	72.41 ± 6.128	97.48 ± 8.809****
Infant weight, g	3144 ± 470.3	2620 ± 703.3**
Placenta weight, g	600.2 ± 105.1	553.4 ± 162.1**

**P < 0.01, **** P < 0.0001.

### RNA-seq and downstream bioinformatics analysis

HTR-8/SVneo cells were transfected with siRNA or negative control for 48 h as described above. Total RNA of three paired samples of HTR-8/SVneo cells was reserved using 1 mL TRIzol (Ambion) and then transferred to Novogene (Beijing). Total RNA was extracted and RNA quality was measured using an Agilent 2100 bioanalyzer. The transcriptome library was constructed by a NEBNext^®^ Ultra™ RNA library prep kit for Illumina^®^; and sequencing was performed on the Illumina Novaseq platform. After quality control, reads were mapped to reference genome using Hisat2. DEseq2 package for R was used for normalisation and to identify differentially expressed genes. Genes with adjusted *P* values less than 0.05 were used to perform enrichment analysis using the ClusterProfiler package for R (Yu *et al.* 2012).

### Statistical analysis

All data were acquired from at least three independent biological replicates. Data were presented as the mean ± s.d., and statistical analysis was performed using GraphPad Prism 8. For parametric data, a Student’s *t*-test was used to compare two groups. For nonparametric data, a Mann–Whitney test was used to compare two groups or a Kruskal–Wallis test was used to compare three or more groups. *P* values < 0.05 were considered statistically significant.

Additional materials and methods are provided in Supplementary Information.

## Results

### CLDN1 was downregulated in the placental villi of patients with preeclampsia

To determine whether certain genes are involved in the pathogenesis of PE, four GEO datasets of the placental transcriptome in preeclampsia (PE) and healthy controls (HCs) were selected (Leavey *et al.* 2016, Than *et al.* 2018, Awamleh *et al.* 2019). Interestingly, two genes were identified based on the intersection of differentially expressed genes of the datasets, including *CLDN1* and *BHLHE40* (Fig. 1A). QRT-PCR was performed to confirm the results of bioinformatics analysis, and the level of *CLDN1* was found to be significantly decreased in the placental villi of PE patients compared to that in HCs (Fig. 1B); however, the level of *BHLHE40* was not significantly different in the present qRT-PCR result; therefore, we chose *CLDN1* for further investigation. Western blotting and immunohistochemical staining were used to confirm a decrease in the expression of *CLDN1* in the placental villus tissue in PE (Fig. 1C and D). Thus, *CLDN1* expression is decreased in trophoblasts of PE patients, suggesting that this decrease may be involved in trophoblast dysfunction.

### Knockdown of CLDN1 inhibits proliferation and induces apoptosis in trophoblasts

**Figure 2:**
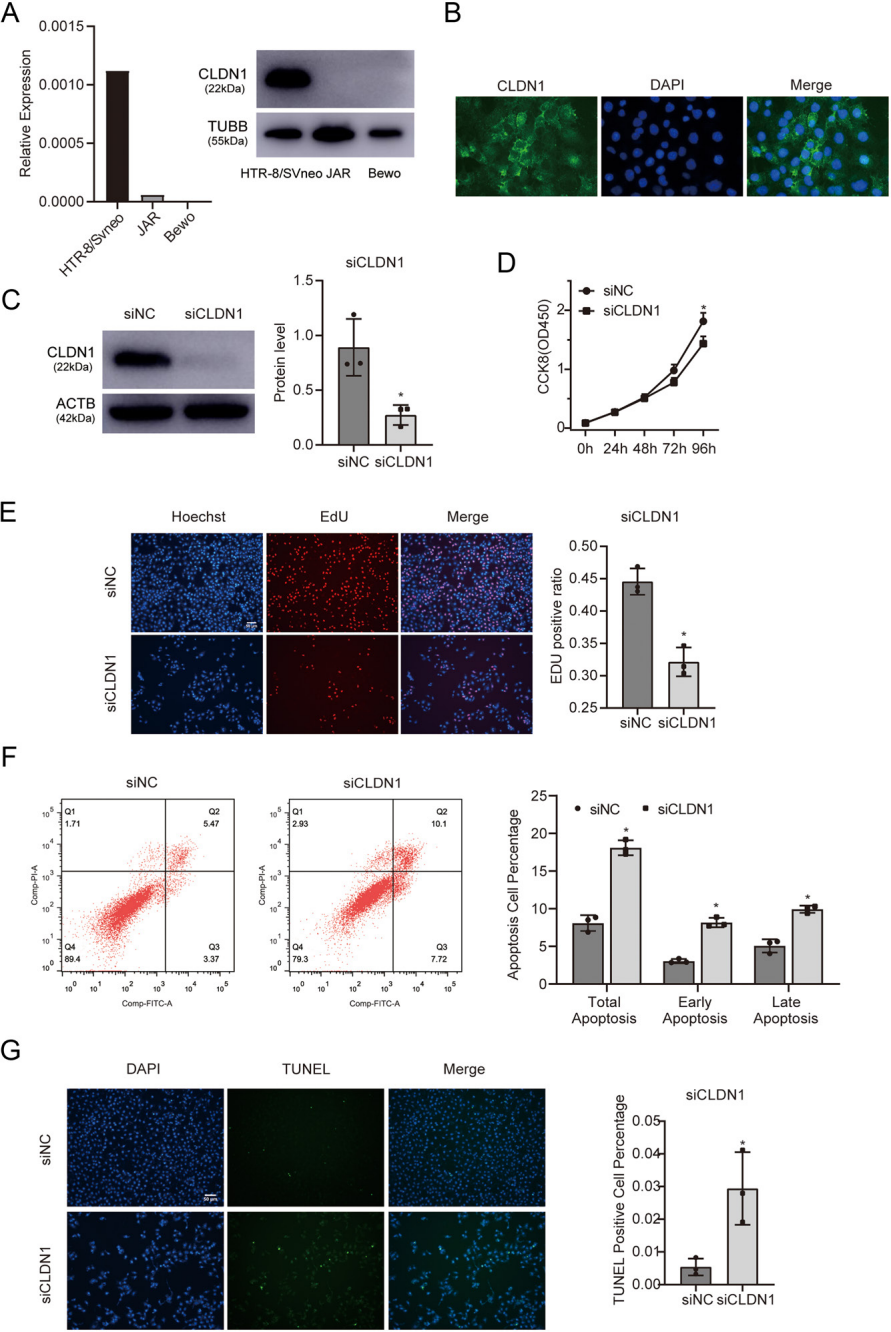
Knockdown of *CLDN1* inhibits the proliferation and induces apoptosis in trophoblasts. (A) *CLDN1* expression levels in three commonly used trophoblast cell lines. (B) Subcellular localisation of *CLDN1* in HTR-8/SVneo cell line. (C) Expression of *CLDN1* after siCLDN1 oligonucleotide administration. (D) CCK-8 assay showing trophoblast proliferation at indicated time points. (E) EdU assay showing the role of *CLDN1* in cell proliferation. (F) Apoptosis was assessed by flow cytometry analysis. (G) TUNEL assay verifying an increase in the proportion of apoptotic cells after *CLDN1* downregulation. Each* in vitro* test test was used, and the data are presented as the mean ± s.d. *P < 0.05; NS, not significant.

To investigate the role of *CLDN1* in PE, we determined the expression level of *CLDN1* in three commonly used trophoblast cell lines, HTR-8/SVneo, JAR and Bewo, using qRT-PCR and Western blotting. The results showed that HTR-8/SVneo had the highest level of *CLDN1* expression (Fig. 2A), and immunofluorescence result showed the subcellular localisation of *CLDN1* in HTR-8/SVneo (Fig. 2B). Therefore, we selected HTR-8/SVneo cells as an *in vitro* model to study the function of *CLDN1* in trophoblasts. We used siRNA to knock down *CLDN1* in HTR-8/SVneo cells, and knockdown efficiency was measured by Western blotting (Fig. 2C). Furthermore, the results of the CCK-8 assay indicated that *CLDN1* knockdown significantly inhibited the proliferation of trophoblasts (Fig. 2D). Accordingly, the EdU assay demonstrated that knockdown of *CLDN1* resulted in a decrease in the proportion of EdU-positive cells, which confirmed that downregulation of *CLDN1* impairs trophoblast proliferation (Fig. 2E). The effect of *CLDN1* on trophoblast apoptosis was evaluated by flow cytometry, and the results showed that knockdown of *CLDN1* promoted trophoblast apoptosis at both early and late stages (Fig. 2F). TUNEL staining confirmed that *CLDN1* knockdown induced apoptosis of the cells (Fig. 2G).

### Overexpression of CLDN1 promotes proliferation and alleviates apoptosis in trophoblasts

**Figure 3:**
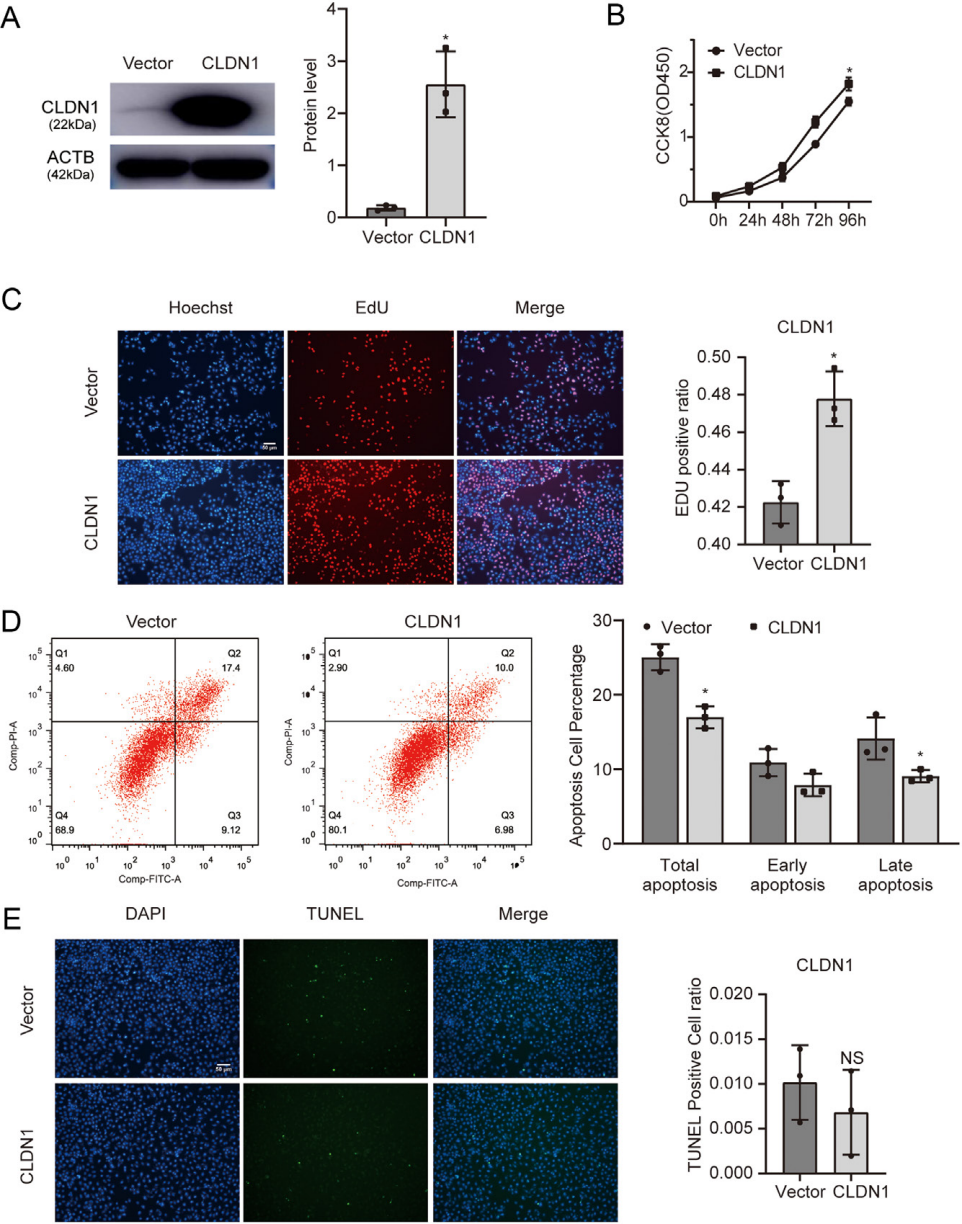
Overexpression of *CLDN1* promotes the proliferation and alleviates apoptosis in trophoblasts. (A) Expression of *CLDN1* after overexpression plasmid administration. (B) CCK-8 assay showing trophoblast proliferation at specific time points. (C) Effects of overexpression of *CLDN1* on cell proliferation determined by EdU assay. (D) The rate of apoptosis assessed by flow cytometry analysis in *CLDN1*-overexpressing trophoblasts. (E) TUNEL assay showed that *CLDN1* upregulation reduced apoptosis in trophoblasts. Each* in vitro* test was performed at least three times, Mann–Whitney test was used, and the data are presented as the mean ± s.d.. *P < 0.05, NS, not significant.

We used a *CLDN1*-overexpressing plasmid to investigate the role of *CLDN1* in trophoblasts. The overexpression efficiency was determined by Western blotting (Fig. 3A). The results of the CCK-8 assay demonstrated an increase in cell viability after *CLDN1* overexpression (Fig. 3B), and the data of the EdU assay also indicated that *CLDN1* overexpression promoted cell proliferation (Fig. 3C). Furthermore, the results of flow cytometry showed that *CLDN1* overexpression decreased the proportion of apoptosis mainly in the late stage (Fig. 3D). Correspondingly, the data of the TUNEL assay showed that *CLDN1* overexpression reduced apoptosis in trophoblasts though not significant (Fig. 3E).

### CLDN1 downregulation affects apoptosis-associated pathways

**Figure 4:**
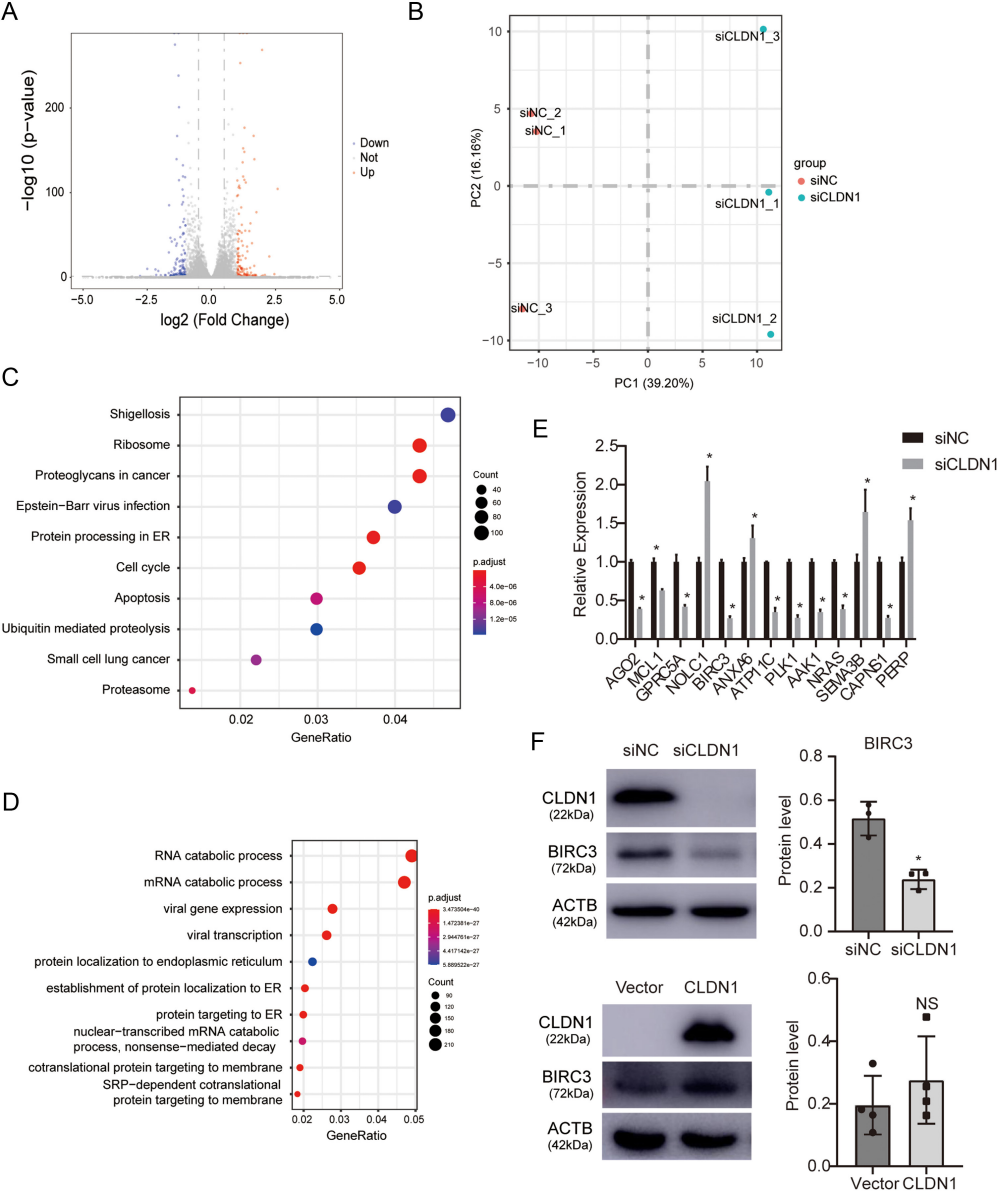
*CLDN1* downregulation affects apoptosis-associated pathways. (A and B) Volcano and heatmap plots showing differentially expressed genes after *CLDN1* knockdown. (C and D) KEGG and GO enrichment analysis. (E) qRT-PCR results of differentially expressed genes after *CLDN1* downregulation. (F) Western blotting assay of BIRC3 after *CLDN1* downregulation or overexpression. Each* in vitro* test was performed three times, Mann Whitney test was used, and the data are presented as the mean ± s.d. *P < 0.05, NS, not significant.

Since *CLDN1* has a significant influence on HTR-8/SVneo apoptosis, we tested apoptosis associated protein BAX and BCL2 in *CLDN1* downregulated cells. However, the results showed that *CLDN1* may not affect apoptosis through these two proteins (Supplementary Fig. 2). RNA-seq assay was used to determine the molecular mechanism of regulation of trophoblast proliferation and apoptosis by *CLDN1*; we identified the differences in the transcriptomes of HTR-8/SVneo cells treated with negative control siRNA (siNC) or siRNA against *CLDN1*. A total of 268 differentially expressed genes were identified, including 144 upregulated and 124 downregulated genes (Fig. 4A and B). Genes with adjusted *P* values less than 0.05 were used to perform the enrichment analysis (Fig. 4C and D). For KEGG analysis, significantly enriched terms included protein processing in endoplasmic reticulum, ubiquitin-mediated proteolysis, apoptosis and cell cycle. Furthermore, qRT-PCR was used to validate the RNA sequencing results of the top 13 differentially expressed genes (Fig. 4E). *BIRC3* was selected for the downstream analysis since it was one of the most prominently differentially expressed genes enriched in the apoptosis pathway. *BIRC3* encodes cellular inhibitor of apoptosis protein 2 (cIAP2), a member of the inhibitors of apoptosis protein family (IAPs), which has been reported to indirectly inhibit caspase activity (Bai *et al.* 2014). Therefore, we detected *BIRC3* protein levels in *CLDN1*-manipulated cells, and the results showed that *BIRC3* was downregulated in *CLDN1* knockdown cells and upregulated in *CLDN1*-overexpressing cells (Fig. 4F). These data indicate that *CLDN1* regulates the expression of *BIRC3* in trophoblasts.

### CLDN1 regulates apoptosis via BIRC3 expression

**Figure 5:**
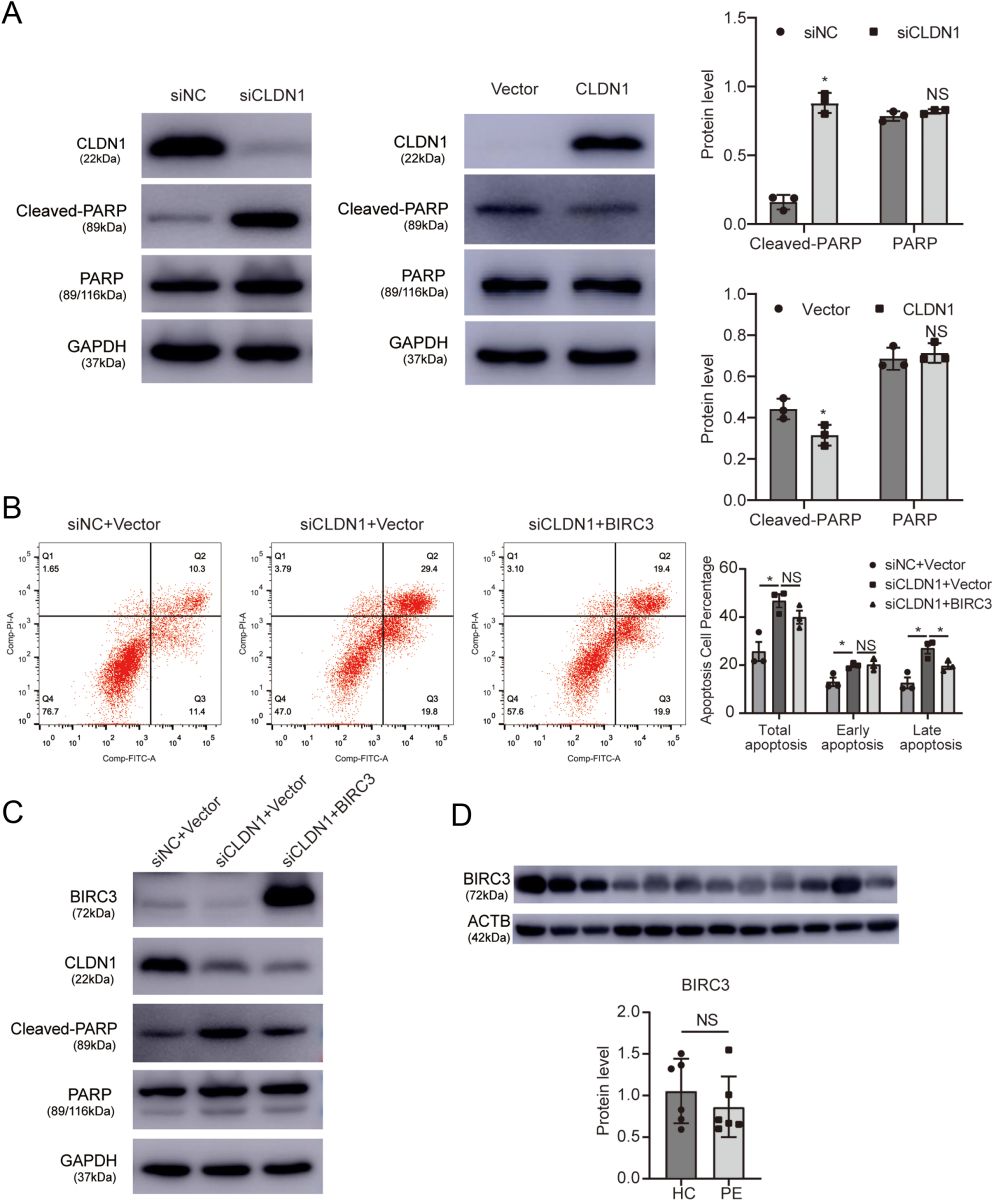
CLDN1 regulates apoptosis through the BIRC3/PARP pathway. (A) Western blotting showing alteration in the levels of cleaved PARP after *CLDN1* downregulation or overexpression. (B) *BIRC3* overexpression in *CLDN1*-downregulated cells reduced trophoblasts apoptosis according to the data of flow cytometry. (C) Level of cleaved PARP after* BIRC3* overexpression in *CLDN1*-downregulated cells. (D) Protein level of BIRC3 in HC and PE. Each* in vitro* test was performed three times, Mann Whitney test were used in (A) and (D) and Kruskal-Wallis test was used in (B), and the data are presented as the mean ± s.d. *P < 0.05, NS, not significant.

Decreased levels of *BIRC3* are commonly accompanied by increased levels of cleaved PARP in apoptotic cells (Zhang *et al.* 2019). Several small molecule mimetics of second mitochondria-derived activator of caspases (SMAC) have been developed to restore apoptosis in the tumour cells (Bai *et al.* 2014, Wang *et al.* 2016). Western blotting was performed to determine whether PARP is involved in trophoblast apoptosis, and the results showed that cleaved PARP was elevated by *CLDN1* downregulation, and the overexpression of *CLDN1* decreased the level of cleaved PARP (Fig. 5A). To verify that the *CLDN1*/*BIRC3*/PARP pathway regulates apoptosis of HTR-8/SVneo cells, we used flow cytometry to analyse the effect of *BIRC3* overexpression on apoptosis after *CLDN1* knockdown (Fig. 5B). The results showed that apoptosis was successfully reduced after *BIRC3* overexpression. In addition, cleaved PARP levels were decreased after overexpression of *BIRC3* in *CLDN1* knockdown HTR-8/SVneo cells (Fig. 5C). Furthermore, *BIRC3* expression was measured in PE and HC villus tissue using Western blotting, and we found that BIRC3 is decreased in the PE placentas compared with HC placentas though the difference is not significant (Fig. 5D). Thus, our results suggested that *CLDN1* inhibited *BIRC3* mRNA levels and that downstream PARP participated in trophoblast apoptosis.

## Discussion

Accumulating studies have demonstrated the transcriptome differences between placenta in preeclampsia (PE) and uncomplicated pregnancy (Nishizawa *et al.* 2007, Awamleh *et al.* 2019). In contrast, this study combined four GEO datasets to compare the transcriptome of placenta in preeclampsia and normal pregnancy using bioinformatics analysis to demonstrate potential differential expression of the *CLDN1* gene. Both the mRNA and protein levels of *CLDN1* were decreased in the placental villi tissue of preeclampsia patients. The tight junction complex plays an important role in cellular activities including proliferation, migration and so on (Garcia-Hernandez *et al.* 2017). As an important protein regulating cell-cell attachment, we assume that the downregulation of *CLDN1* to some extent disrupted the completeness of plasma membrane which is vital to maintain the integrity of the cell function. In this article, we confirmed that a decrease in *CLDN1* leads to the induction of apoptosis in trophoblasts.

Increased apoptosis of villus trophoblasts in placenta pathologies has been observed in many pregnancy-associated diseases, including pregnancy loss, preeclampsia and intrauterine growth restriction (Ishihara *et al.* 2002, Sharp *et al.* 2010). Hypoxia-reoxygenation and oxidative stress are associated with trophoblast apoptosis (Soleymanlou *et al.* 2007). Moreover, Kalkat * et al.* reported that the balance of BCL2 family proteins was altered towards cell death in preeclampsia (Kalkat *et al.* 2013). Recent studies have focused on trophoblast apoptosis caused by molecular dysfunction that leads to the disease (Guo *et al.* 2017, Wang & Yan 2018, Mo *et al.* 2020). *CLDN1* has been shown to be involved in apoptosis via alterations in the expression levels of Bcl-2 and Bax (Singh *et al.* 2012, Zheng *et al.* 2019), which was not detected in our study (Supplementary Fig. 2). We suggest that this difference may be due to the heterogeneity of tissue origin.

The impact of the *CLDN1* gene on apoptosis has been investigated in a number of studies. For example, *CLDN1* downregulation was shown to promote apoptosis and hinder cell proliferation in tumour cells (Huang *et al.* 2015, Zheng *et al.* 2019). Some studies demonstrated that *CLDN1 was* associated with the activation of autophagy (Zhao *et al.* 2017), and other studies concluded that *CLDN1* regulates anoikis (Singh *et al.* 2012, Huang *et al.* 2015). Our data indicated that downregulation of *CLDN1* inhibited the proliferation and induced apoptosis in trophoblasts. Since the detailed mechanism by which *CLDN1* regulates trophoblast apoptosis is unclear, we used RNA-seq in *CLDN1*-knockdown HTR-8/SVneo cells. The results identified *BIRC3* as a molecule downstream of *CLDN1*. *BIRC3* was shown to be involved in the regulation of both intrinsic and extrinsic apoptosis pathways via indirect regulation of caspase activity (Varfolomeev *et al.* 2007, Bai *et al.* 2014, Gressot *et al.* 2017, Lee *et al.* 2017, Zhang *et al.* 2019). Majority of claudins contain a PDZ binding motif that interacts with intercellular molecules that have signalling functions (Garcia-Hernandez *et al.* 2017). As a tight junction protein, claudin-1 was confirmed to be associated with Src/p-Src in colon cancer cells in modulating cell anoikis (Singh *et al.* 2012). It was also found that claudin-1 regulates cell anoikis through ZEB-1 (Singh *et al.* 2011). Evidence has also been found that claudin-1 regulates apoptosis in human breast cancer cells through deactivation of pro-caspase-8 (Liu *et al.* 2012). Other claudin family member such as claudin-8 interacts with IL22 in colon cell lines to rescue apoptosis induced by LINC00662 knockdown (Cheng *et al.* 2020). However, the mechanism of how *CLDN1* regulates downstream *BIRC3* remains to be determined.

Accumulated cleaved PARP has been detected in other trophoblast cell lines in a study that demonstrated that increased apoptosis in trophoblasts is accompanied by an increase in the levels of proapoptotic proteins (Chen *et al.* 2015). We hypothesised that the accumulation of cleaved PARP caused by *BIRC3* downregulation may be involved in the mechanism of PE pathogenesis. Thus, we investigated the effect of *CLDN1* knockdown on the level of cleaved PARP. The results showed that knockdown of *CLDN1* increased the level of cleaved PARP, and subsequent experiments showed that overexpression of *BIRC3* in *CLDN1*-downregulated HTR-8/SVneo cells reduced cell apoptosis. Some studies showed associations between *BIRC3* and necrosis (Geserick *et al.* 2009, Bai *et al.* 2014); hence, we examined the impact of *CLDN1* downregulation on trophoblast necrosis. However, the levels of phosphorylated RIP and phosphorylated MLKL showed trends that were not consistent with this assumption (Supplementary Fig. 2).

**Figure 6:**
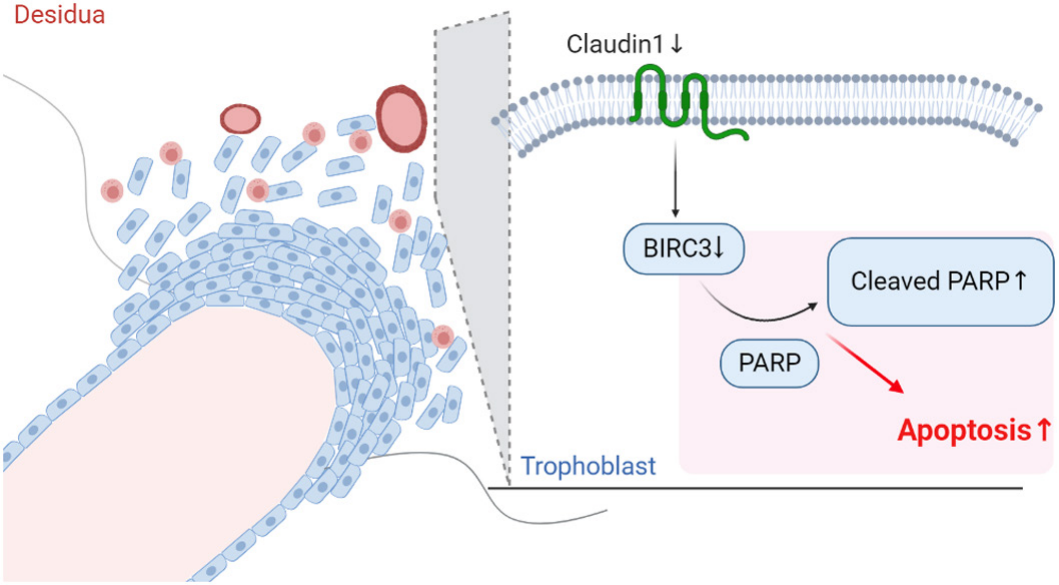
Schematic depiction of *CLDN1* in trophoblast apoptosis. (Created with BioRender.com).

In conclusion, we demonstrated that downregulation of *CLDN1* regulates trophoblast apoptosis via a decrease in the levels of *BIRC3* and downstream PARP, which is a new phenomenon in the pathogenesis of preeclampsia (Fig. 6). Our study demonstrated the association of *CLDN1* and preeclampsia, which can offer a new potential therapeutic target for future clinical applications. However, current results are based on experiments of trophoblast cell lines which is still quite different to the normal trophoblasts; and further investigation using primary trophoblast cells may help to further verify our conclusion.

## Supplementary Material

Supplementary Figure 1 PCA analysis of the four GSE datasets used in this study. (A) Six PE and five Healthy controls (HCs) were selected in GSE66273 datasets. (B)Three preeclampsia (PE) and Four HCs were selected in GSE96984 datasets. (C)Eighty-four PE and thirty-three HCs were selected in GSE75010 datasets. (D) Twenty PE and Twenty-one HCs were selected in GSE114691 datasets.

Supplementary Figure 2 Western blotting results of some apoptosis and necrosis associated proteins. (A) Western blotting results of BAX in CLDN1 knockdown HTR-8/SVneo. (B) Western blotting results of BCL2 in CLDN1 knockdown HTR-8/SVneo. (C) Western blotting results of phosphorylated RIP(P-RIP) and phosphorylated MLKL(P-MLKL) in CLDN1 knockdown HTR-8/SVneo. (D) Western blotting results of RIP and MLKL in CLDN1 knockdown HTR-8/SVneo. Each in vitro test was performed three times, Mann Whitney test was used, and the data are presented as the mean ± SD. *P < 0.05, NS, not significant.

Supplementary Information

Supplementary Table S1 Primers and antibodies used in this study

Table S2 Clinical information of patients included for three experiments respectively

## Declaration of interest

The authors declare that there is no conflict of interest that could be perceived as prejudicing the impartiality of the research reported.

## Funding

This work was supported by the National Key Research and Development Program of China (2018YFC1002800 to Yan Zhang and Yi Lin), the Special Funds for Local Science and Technology Development Guided by the Central Committee (2018ZYYD014 to Yan Zhang), the National Natural Science Foundation of China (81971403 to Yi Lin), the National Natural Science Foundation of China (82071647 to Fuju Tian), the National Natural Science Foundation of China (81671519 to Fengtao Shi) and the Clinical Research Fund of the International Peace Maternity and Child Health Hospital, Shanghai Jiao Tong University School of Medicine (GFY5816 to Yi Lin). The authors would like to thank the Department of Biobank (International Peace Maternity and Child Health Hospital, Shanghai Jiao Tong University) for providing clinical samples.

## Author contribution statement

Y-C Z and F-J T conceived and designed the experiments. Y-C Z, X-L Q, H-Q M, C-X Z helped to collect samples, Y-C Z, H-Q M, S Q, X-W W and X-Q L helped to perform the experiments. Y-C Z and F-J T analysed the data and wrote the manuscript, F-J T, Y Z and Y L contributed reagents and materials and to revise the manuscript.
