# Supplementary figures and images for: CLDN1 regulates trophoblast apoptosis and proliferation in preeclampsia
Source: Reproduction. 2021 Mar 30;161(6):623–32. doi: 10.1530/REP-20-0677 (PMC8111329; doi:10.1530/REP-20-0677)

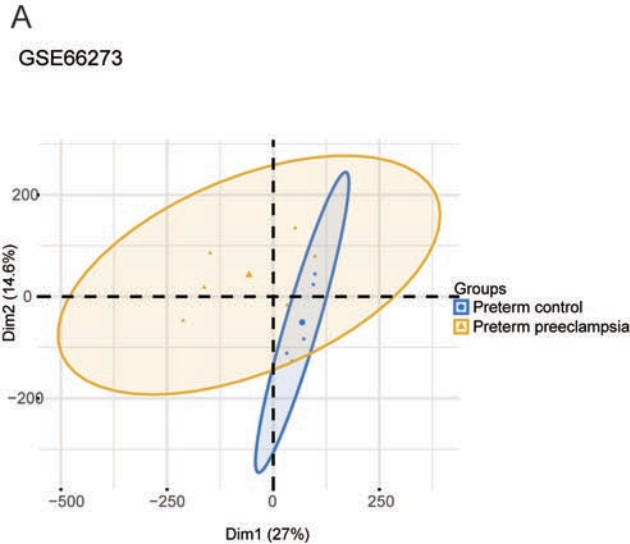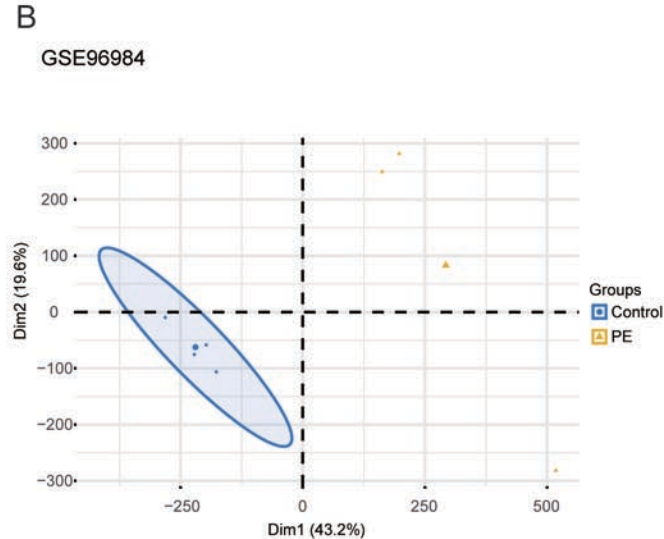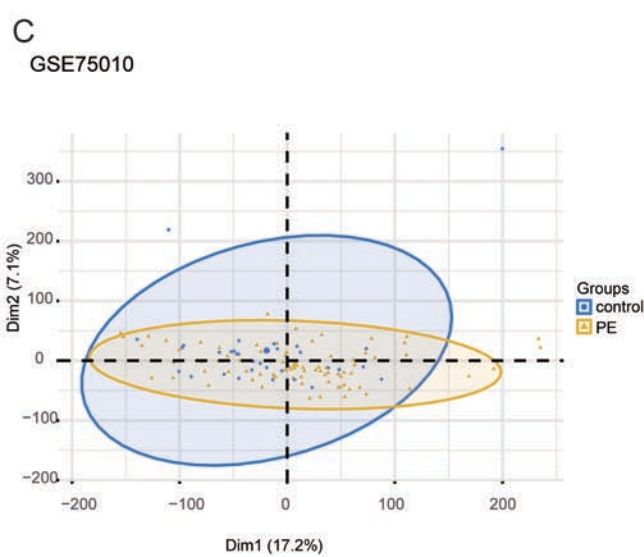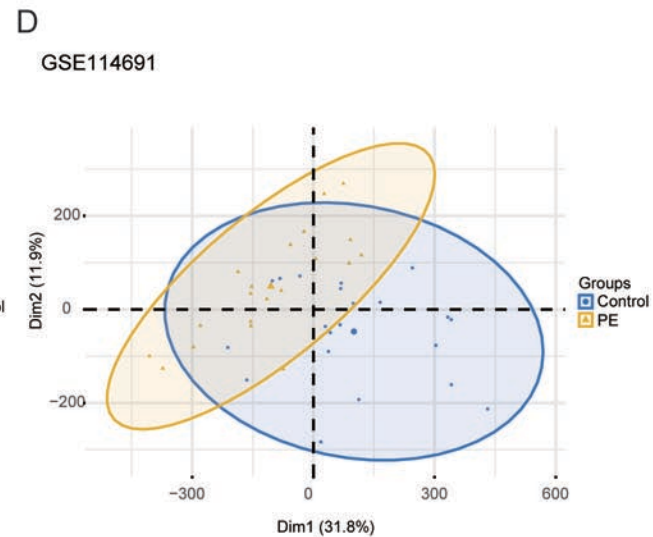

Supplement: Supplementary Figure 1 PCA analysis of the four GSE datasets used in this study. (A) Six PE and five Healthy controls (HCs) were selected in GSE66273 datasets. (B)Three preeclampsia (PE) and Four HCs were selected in GSE96984 datasets. (C)Eighty-four PE and thirty-three HCs were selected in GSE75010 [file supplementary_figure_1.pdf]

A

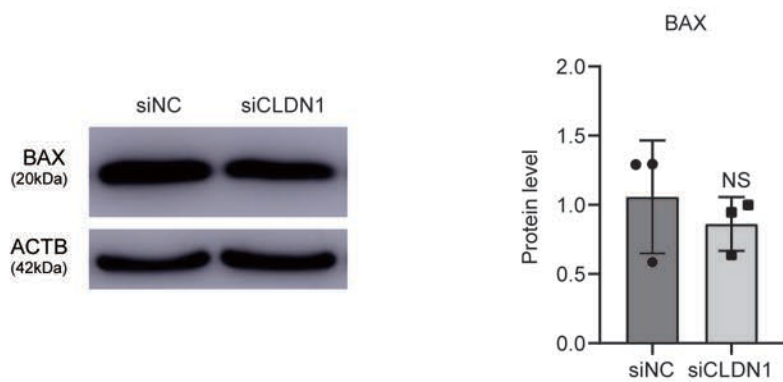

B

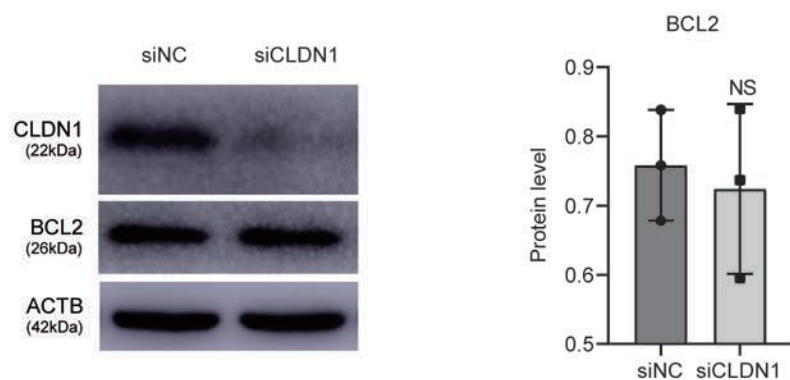

C

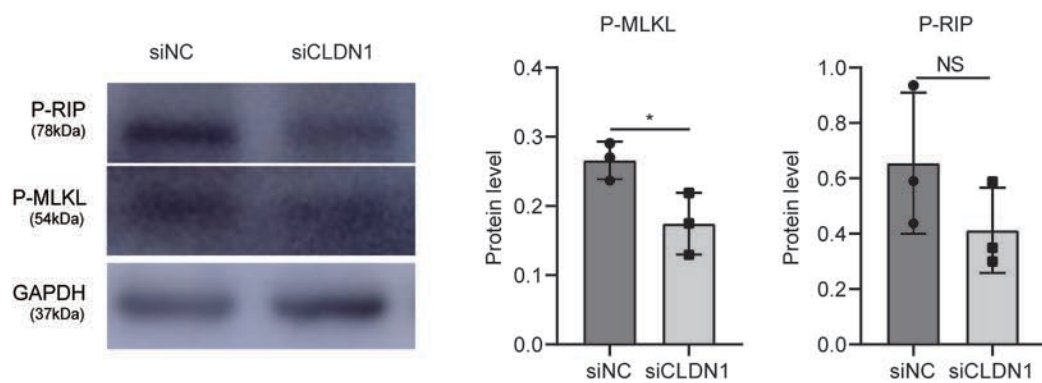

D

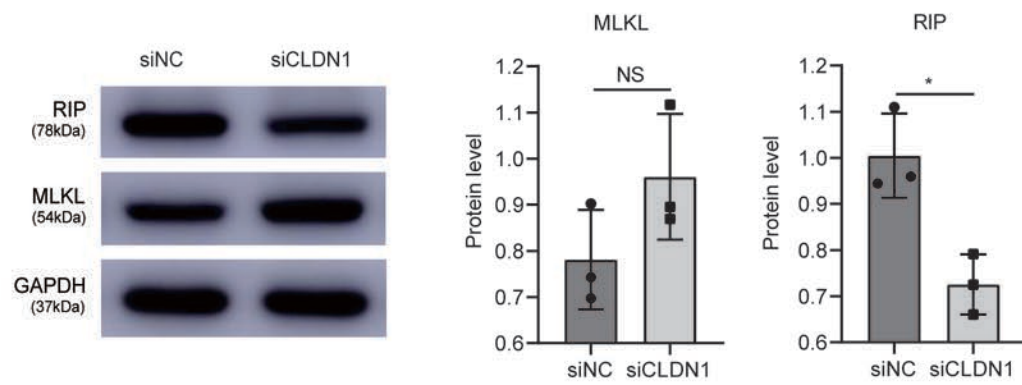

Supplement: Supplementary Figure 2 Western blotting results of some apoptosis and necrosis associated proteins. (A) Western blotting results of BAX in CLDN1 knockdown HTR-8/SVneo. (B) Western blotting results of BCL2 in CLDN1 knockdown HTR-8/SVneo. (C) Western blotting results of phosphorylated RIP(P-RIP) and p [file supplementary_figure_2.pdf]
