# Supplementary material for: CLDN1 regulates trophoblast apoptosis and proliferation in preeclampsia
Source: Reproduction. 2021 Mar 30;161(6):623–32. doi: 10.1530/REP-20-0677 (PMC8111329; doi:10.1530/REP-20-0677)
Supplement: Supplementary Information [file supplementary_material.pdf]

Supplementary Information

Supplementary Figure 1 PCA analysis of the four GSE datasets used in this study. (A) Six PE and five Healthy controls (HCs) were selected in GSE66273 datasets. (B)Three preeclampsia (PE) and Four HCs were selected in GSE96984 datasets. (C)Eighty-four PE and thirty-three HCs were selected in GSE75010 datasets. (D) Twenty PE and Twenty-one HCs were selected in GSE114691 datasets.

Supplementary Figure 2 Western blotting results of some apoptosis and necrosis associated proteins. (A) Western blotting results of BAX in *CLDN1* knockdown HTR-8/SVneo. (B) Western blotting results of BCL2 in *CLDN1* knockdown HTR-8/SVneo. (C) Western blotting results of phosphorylated RIP(P-RIP) and phosphorylated MLKL(P-MLKL) in *CLDN1* knockdown HTR-8/SVneo. (D) Western blotting results of RIP and MLKL in *CLDN1* knockdown HTR-8/SVneo. Each in vitro test was performed three times, Mann Whitney test was used, and the data are presented as the mean ± SD. *$P < 0.05$, NS, not significant.

Sample collection

Placental tissue for RNA extraction was initially stored in RNAlater (Invitrogen) at 4°C overnight before moving into long-term preservation at -80°C. Tissue for protein extraction was immediately stored at -80°C. For immunohistochemistry, tissue was fixed in 4% paraformaldehyde (BBI) for 24 h and then transferred into 75% alcohol before paraffin embedding. Some of the samples were obtained from the biobank of the International Peace Maternity & Child Health Hospital. All the experiments were modified in the same way in the two groups.

RNA extraction, reverse transcription and quantitative real-time PCR

Total RNA was extracted from placental villus tissue or cell lines using Trizol reagent (Invitrogen). Concentration and quality of the total RNA were tested using Nanodrop 2000 (Thermofisher Scientific). RNA reverse transcription into cDNA was performed using PrimeScript™ RT reagent Kit with gDNA (Takara). Primers used in this experiment were listed in Supplementary Table S1. Gene expression level was measured using an SYBR green kit (Takara), conditions are set as follows: 95°C 30s for pre-degeneration; 40 cycles of denaturation: 95°C (5s), followed by annealing:60°C(30s). The relative gene expression level was calculated by the formula: $2^{-(\Delta Ct\ \text{target gene}\ -\Delta Ct\ \text{ACTB})}$.

Protein extraction and western blotting

Proteins were obtained using RIPA Lysis and Extraction Buffer (Thermo Scientific), concentrations of tissue protein were measured by Pierce™ BCA Protein Assay Kit (Thermo Scientific), cell protein lysates were collected in 1× Protein Loading Dye (Sangon Biotech). After boiling at 95℃ for 10 min, around 20 μg linear proteins were separated by 10-12.5% of sodium dodecyl sulfate PAGE (SDS-PAGE) and transferred onto polyvinylidene difluoride (PVDF; BioRad Laboratories) membranes. The membranes were blocked under room temperature with 5% non-fat milk (Beyotime Biotechnology) for 1-2 h, and then incubated with specific primary antibodies under 4℃ overnight. Washed with Tris buffered saline-Tween for three times, the membranes were incubated with corresponding second antibodies at room temperature for 1-1.5 h. Protein bands were visualized by enhanced chemiluminescence

(Millipore). Intensities of bands were evaluated by ImageJ (National Institutes of Health, USA). Antibodies used were listed in Supplementary Table S1.

Immunohistochemistry (IHC)

A mouse and rabbit specific HRP/DAB (ABC) detection IHC kit was used (ab64264, Abcam). Paraffin-embedded tissue was sectioned into 5 μm paraffin sections. Tissue sections were deparaffinized, rehydrated and boiled in 1× citrate-EDTA buffer for antigen retrieval. Hydrogen Peroxide Block and Protein Block were applied before incubating with primary antibodies (anti-human *CLDN1*, 1:250 dilution, Abcam) at 4°C overnight. Biotinylated Goat Anti-Polyvalent and Streptavidin Peroxidase were incubated for 10 min at room temperature. Incubation time of DAB was kept the same for all sections. After counterstain and dehydrate, tissue sections were sealed. Images were captured using a Leica DMi8 microscope (Leica Microsystems). Staining intensity was evaluated by Image-Pro Plus 6.0 software.

Cell culture

The HTR-8/SVneo cell line was a gift of Dr PK Lala (University of Western Ontario). Cells were cultured in Dulbecco's modified Eagle's medium:nutrient mixture F-12 (DMEM/F-12, HyClone) containing 10% foetal bovine serum (Gibco) in an atmosphere containing 5% CO_2 at 37°C. Culture medium was changed every 2 or 3 days. Trypsin (0.25%, w/v) was used to detach the cells at 37°C for 3 min after complete removal of the serum.

Small-interfering RNA knockdown

Approximately 3.0×10^6 cells were cultured in a well of a 6-well plate with complete growth medium overnight to achieve approximately 40% confluence at the time of transfection. Three different siRNAs designed for *CLDN1* were purchased from GenePharma (Shanghai,

China) and mixed to develop a pool of siRNAs (si*CLDN1*-1, (sense)5'-GCAAAGUCUUUGACUCCUUTT-3'; (antisense)5'-AAGGAGUCAAAGACUUUGCTT-3'; si*CLDN1*-2, (sense)5'-CCACAGCAUGGUAUGGCAATT-3'; (antisense)5'-UUGCCAUACCAUGCUGUGGTT-3'; si*CLDN1*-3, (sense)5'-GGUGCCCUACUUUGCUGUUTT-3'; (antisense)5'- AACAGCAAAGUAGGGCACCTT-3'; nontargeting control siRNA, (sense)5'-UUCUCCGAACGUGUCACGUTT-3'; (antisense)5'-ACGUGACACGUUCGGAGAATT-3'). Three microlitres of Oligofectamine transfection reagent (Invitrogen) was incubated with 7.5 μL of siRNA for 10 min at room temperature, and the mixture was added to the cells in serum-free culture medium to a final concentration of 75 nM. The same volume of the culture medium containing 20% FBS was added 4-6 h after the transfection.

Plasmid overexpression

A *CLDN1* overexpression plasmid was constructed by cloning the coding region sequence (CDS) of human *CLDN1* into the pEX-3 vector (GenePharma Shanghai, China). Approximately 5.0×10^6 cells were cultured in a well of a 6-well plate in complete growth medium overnight to achieve approximately 60% confluence at the time of transfection. A total of 1.5 μg of the plasmid was mixed with 3 μL of JetPRIME® reagent (Polyplus-transfection® SA, Strasbourg) in 200 μL of JetPRIME buffer. After incubation at room temperature for 10 min, the mixture was added to the cells in 800 μL complete culture medium, and the samples were incubated for 4-6 h.

A *BIRC3* overexpression plasmid was constructed by VectorBuilder (China); overexpression of *BIRC3* was conducted 24 hours after siRNA transfection. Further experiments were performed 48 hours after *BIRC3* plasmid transfection.

Cell counting kit-8 assay

Twenty-four hours after the transfection, the cells were digested and counted; 4×10^3 cells per well were plated in 96-well plates, and 6 replicates were tested in each group. Cell viability was assessed using a cell counting kit-8 (YEASEN, China) at 0, 24, 48, 72 and 96 h.

EdU assay

Forty-eight hours after the transfection, the cells were incubated with 50 μM EdU for 2 h at 37°C. Then, the cells were fixed with 4% paraformaldehyde and permeabilized with 0.5% Triton X-100. Fluorescent staining was performed according to the manufacturer's instructions (RiboBio). A Leica DMi8 microscope was used to detect the signal (Leica Microsystems).

TUNEL assay

Forty-eight hours after the transfection, the cells were fixed with 4% paraformaldehyde and permeabilized with 0.5% Triton X-100. After equilibration with 1× equilibration buffer for 10 min at room temperature, the cells were fluorescently stained using a terminal deoxynucleotidyl transferase nick-end-labelling (TUNEL) reaction mixture containing TdT and Alexa Fluor 488-12-dUTP (YEASEN, China). DAPI solution (2 μg/mL) was added for 5 min at room temperature. Images were captured using a Leica DMi8 microscope (Leica Microsystems).

Flow cytometry analysis for apoptosis

Forty-eight hours after the transfection, the cells were digested and counted; approximately 5×10^5 cells were washed twice with PBS and gently resuspended in 100 μL of 1× binding buffer (BD) containing 5 µl FITC Annexin V and 5 µl PI. After incubation at room temperature in the dark for 15 min, another 400 μL of 1× binding buffer was added. Flow cytometry analysis was carried out within 1 h.
